# Perceptual learning of detection of textures in noise

**DOI:** 10.1167/jov.20.7.22

**Published:** 2020-07-21

**Authors:** Zahra Hussain, Patrick J. Bennett

**Affiliations:** 1 Department of Psychology, American University of Beirut, Beirut, Lebanon; 2 Department of Psychology, Neuroscience and Behaviour, McMaster University, Hamilton, Ontario, Canada

**Keywords:** sensitivity, bias, noise, channel, stimulus specific, transfer, generalization

## Abstract

We examined whether the effects of practice on visual detection are stimulus specific and whether practice alters response bias. Eighty-four subjects performed yes-no detection of band-limited noise patterns (textures) in two levels of external noise, on two consecutive days. On day 2, one-half of the observers switched to novel textures. Texture contrast was varied on signal-present trials using the method of constant stimuli. The signal was present on 50% of the trials. We measured *d*^′^, detection thresholds, and two measures of response criterion: a global criterion that was based on sensitivity at all signal levels (Jones et al., 2015) and a local criterion computed at a hit rate of 70% or 80% (Wenger & Rasche, 2006). Performance improved for both groups on day 2, indicating that improvement transferred to novel textures. Increases in *d*^′^ were associated with a decrease in false alarms across days. The global criterion became less liberal and became more optimal (i.e., less biased) with practice; however, this effect was small and was not statistically significant in all conditions. The local criterion measure also became slightly less liberal with practice in most conditions, becoming more or less optimal depending on the hit rate at which it was computed. Overall, the effects of practice on sensitivity in a visual detection task generalized to novel patterns. In addition, we found that practice had relatively small effects on response criterion, and the precise effects on response bias differed between global and local measures of criterion.

## Introduction

The effects of practice on performance in visual discrimination and identification tasks frequently are specific to the trained stimuli. For example, improvements often are largely abolished when stimuli are rotated or moved to a different location in the visual field, or when the task is performed with novel items from the trained stimulus class (Fiorentini & Berardi, 1981; Fahle et al., 1995; Schoups et al., 1995; Crist et al., 1997; Hussain et al., 2009, 2011, 2012). Stimulus-specific effects in discrimination and identification tasks may arise from observers learning to use particular, informative spatial features to discriminate stimuli (Gold et al., 2004; Li et al., 2004; Kurki and Eckstein, 2014). Consequently, the resulting perceptual learning is diminished when the patterns are rotated or reversed in contrast (Hussain et al., 2009). Here, we consider the effects of learning in a visual detection task, in which stimulus features are not clearly visible on most trials. In this situation, it is unlikely that observers could learn to use a *particular* feature to perform the task. Therefore, the effects of learning might generalize to stimuli that share the general spatial characteristics of the signal regardless of feature location, size and other attributes. We examined this possibility for detection of textures in noise. Observers practiced yes-no detection of band-limited noise patterns (textures), in two levels of Gaussian noise over 2 consecutive days, with one-half of the observers switching to new textures on day 2. We examined whether improvements in detection transferred to novel textures.

Previous work examining perceptual learning of detection of simpler patterns such as sine-wave gratings and Gabors patterns has produced mixed findings. For example, Sowden et al. (2002) found that learning of grating detection generalized across stimulus orientation, but not across retinal location or changes in spatial frequency of more than 1.5 octaves. Mayer (1983) reported that learning to detect oblique gratings partially transferred to orthogonal orientations but not to cardinal orientations for which thresholds were presumably already at floor. Practice with detection of cardinal orientations did not benefit detection of oblique orientations (Mayer, 1983). Dorais and Sagi (1997) reported that decreases in Gabor contrast masking thresholds with practice were specific to the orientation and phase of the targets and to the phase of the mask. Therefore, improvements in detection occasionally generalize across orientation, but may be tuned to properties such as spatial frequency and phase.

An additional goal of this work was to examine practice effects on response bias. Some evidence suggests that improvements in perceptual performance are affected by changes in response bias (Wenger & Rasche, 2006; Wenger et al., 2008; Aberg & Herzog, 2012; Jones et al., 2015). For instance, threshold improvements in a visual contrast detection task were accompanied by liberal shifts in the response criterion, decreasing the improvement that might have been achieved otherwise (Wenger and Rasche, 2006). In another study that used an auditory detection task, participants became less liberal with practice, and the decreases in bias were correlated with performance improvements (Jones et al., 2015). We measured sensitivity (*d*^′^) and response bias to assess whether criterion shifts could account for learning in this yes-no detection task, and whether such shifts varied with stimulus novelty.

## Methods

### Subjects

Eighty-four subjects between the ages of 18 and 38 years (*M* = 21.1 years) took part in the experiment either for remuneration ($10/hour) or for course credit. Fifty-three subjects were female. Subjects were students and staff at the University of Nottingham, UK, and McMaster University, Canada. The Nottingham subjects were run first (n = 24; December, 2013). The experiment was then replicated twice at McMaster University, first in March, 2014 (n = 29) and then in October, 2019 (n = 31). Seven subjects (one from Nottingham, two from the first replication at McMaster, and four from the second replication) were excluded from the analyses (see details in Results). Hence, the total sample size was 77 subjects. All subjects had normal or corrected-to-normal visual acuity as measured by the Early Treatment Diabetic Retinopathy Study (ETDRS) visual acuity chart.

### Apparatus and stimuli

Stimuli were generated on a Power Mac G4 computer using Matlab (The Mathworks, version 5.2.1) and the Psychophysics and Video Toolboxes (Brainard, 1997; Pelli, 1997). Stimuli were displayed on CRT monitors (McMaster: Sony Trinitron GDM-F520; Nottingham: Trinitron Dell P1130) set to a resolution of 1280 × 1024 pixels and a frame rate of 85 Hz (non-interlaced). Average luminance was 73 cd/m^2^ (McMaster), and 52.4 cd/m^2^ (Nottingham). The monitor calibration data were used to build a 1,779-element lookup table (Tyler et al., 1992), and customized computer software constructed the stimuli on each trial by selecting the appropriate luminance values from the calibrated lookup table and storing them in the display's eight-bit lookup table.

**Figure 1.**
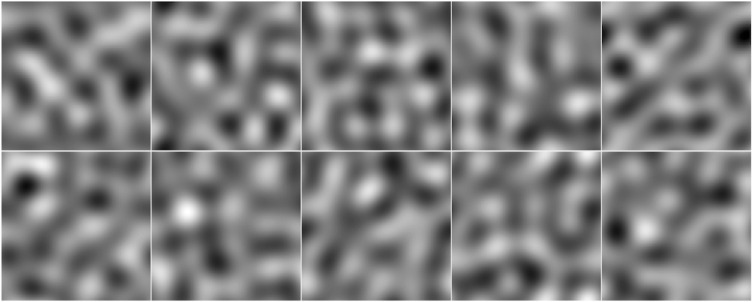
Texture stimuli. Each texture was created by applying an isotropic, bandpass (2-4 cy/image) ideal spatial frequency filter to Gaussian white noise. Top row shows textures comprising set A. Bottom row shows textures comprising set B.

The textures were band-limited noise patterns created by applying an isotropic band-pass (2-4 cycles/image) ideal spatial frequency filter to Gaussian noise (see Figure 1). The textures subtended 4.8^○^ × 4.8^○^ of visual angle from the viewing distance of 114 cm. The stimuli were shown in one of two levels of two-dimensional static, white Gaussian noise created by sampling from distributions with contrast variances of 0.01 and 0.1. During the experiment, signal contrast in each noise condition was varied across trials using the method of constant stimuli. For the signal present condition, the signal was shown at one of seven levels of contrast spaced equally on a logarithmic scale. For the signal absent condition, signal contrast was set to zero. Hence, there were eight contrast levels in all, and 16 different stimulus conditions (eight contrasts × two external noise levels). Table 1 gives the contrasts used for each of the two noise levels. Two sets of five textures were created, set A and set B.

**Table 1.** RMS contrasts used in the detection tasks in both noise levels. Zero contrast corresponds with the signal absent condition, comprising 50% of trials.

Low Noise (0.01)	High Noise (0.1)
0.00	0.00
0.0038	0.0092
0.0043	0.0106
0.0050	0.0122
0.0058	0.0141
0.0066	0.0162
0.0076	0.0187
0.0088	0.0216

### Procedure

All subjects performed two sessions of a yes-no detection task at roughly the same time on two consecutive days. There were two groups. The *same texture* group performed the task with the same set of five textures on both days (either set A or set B on both days). The *different texture* group performed the task with a set of five textures on day 1 and a different set of five textures on day 2 (A-B or B-A on days 1 and 2). The textures are shown in Figure 1.

Viewing was binocular and head position was stabilized with an adjustable chin rest. The stimulus display was the only source of illumination in the room. The experiment started after a 60 s period during which the subject adapted to the average luminance of the display. Each trial began with the presentation of a black, high-contrast fixation point (0.15^○^ × 0.15^○^), in the center of the screen for 100 ms. This was followed by a randomly selected texture presented for 200 ms at the center of the screen in either the signal absent condition (zero contrast) or the signal present condition (one of seven contrasts shown in Table 1) at the given noise level. On signal absent trials, the stimulus comprised a square patch of Gaussian noise. After the stimulus disappeared, subjects used a keypress to report whether the texture was present or absent on that trial. Auditory feedback indicated whether the response was correct (high-pitched tone) or incorrect (low-pitched tone). The next trial began 1 s after the presentation of feedback. Noise levels were blocked with a short break between blocks. The order of noise levels was randomized for each subject. Each session comprised 560 trials: for each noise level there were 140 signal present trials (7 contrasts × 20 trials per contrast), and 140 signal absent trials (zero contrast). Hence, the signal was present on 50% of trials. The duration of each session was about 40 minutes.

Before the experiment began, subjects were shown examples of the stimuli in low and high noise, at both low and high signal contrasts. They were told that the noise conditions would be blocked. They were further given the following instructions: “When you respond correctly, you will hear a high-pitched beep, and when you respond incorrectly, you will hear a low-pitched beep, so you will know how you are doing. Don't feel discouraged if you find the task hard at first, you'll get the hang of it as you go along. The task is designed to be difficult, so you will get some trials wrong from time to time, but please try to do your best.” In this sense, subjects were instructed to maximize the number of correct responses. However, subjects were not explicitly instructed about differences in potential task strategies, that is, “minimize false alarms” vs. “maximize proportion correct.”

## Results

We calculated the signal detection measure of sensitivity (*d*^′^) at each contrast using the standard formula

(1) $$d^{'}=z\left(H\right)-z\left(\mathrm{FA}\right)$$

where *z* is the inverse of the cumulative normal distribution function and *H* and FA are the hit and false alarm rates, respectively. Hits were measured separately at each contrast at each noise level, and a single false alarm rate was measured at each noise level from the signal-absent trials in that block. Contrast detection thresholds corresponding to a *d*^′^ of 1 were obtained from a linear fit of *d*^′^ to log contrast variance.

Response criterion was measured in two ways. The first measure, termed c_local was calculated using the procedure described by Wenger and Rasche (2006), who used the method of constant stimuli for a contrast detection task performed on multiple days. They fit a psychometric function to the proportion of hits measured on day 1 to estimate the stimulus contrast corresponding to a hit rate of 79% (*C*_*H*79_), and then used the false-alarm rate (estimated from the signal-absent trials) to calculate the response criterion using the formula

(2) c=-0.5×[z(H)+z(FA)].

On each subsequent day of testing, a psychometric function that was fit to the hit rates measured with all contrasts was used to estimate the hit rate for the threshold contrast *C*_*H*79_ calculated on day 1. Typically the hit rates for that contrast increased as a result of learning. Finally, Equations 1 and 2 were used to calculate *d*^′^ and *c* from the estimated hit rate and the false-alarm on each day. This procedure yielded estimates of *d*^′^ and *c* for a single contrast (*C*_*H*79_) on every day of testing. We used this procedure to compute *d*^′^ and c_local for stimulus contrasts that produced hit rates of 70% and 80% on day 1 ($c\_{\mathrm{local}}_{70}$ and $c\_{\mathrm{local}}_{80}$). Note that *d*^′^ and *c* on day 1 are determined by a subject's false-alarm rate because the stimulus contrast always was selected to produce a hit rate of either 70% or 80%. On day 2, *d*^′^ and *c* were determined by the false-alarm rate and an estimate of the hit rates at the same contrasts that were used to calculate sensitivity and response bias on day 1.

The second measure of criterion, c_global, was based on Jones et al's (2015) formulation of criterion, which accounts for sensitivity at multiple signal levels:

(3) $$\begin{array}{c} c\_global=\lambda \_obs-\lambda \_ideal\;\; \end{array}$$

(4) $$\begin{array}{ccc} \; & = & -Z\left(FA\right)-\arg\max_{\lambda }\left(\sum_{i=1}^{m}(P\left(S_{i}\right)\left[1-\Phi \left(\lambda ;d_{i}^{'},1\right)\right]\right)\\ \; & & +P(N)[\Phi (\lambda ;0,1)]) \end{array}$$

where *P*(*S*_*i*_) is the probability of the *i*th signal (i.e., 0.5/7), *P*(*N*) is the probability of a noise trial (i.e., 0.5), Φ is the cumulative Gaussian thresholded at the ideal criterion, λ, and assuming equal, unit variance for both distributions. The ideal criterion is assumed to maximize proportion correct for the ensemble of stimulus contrasts. Equation 4 finds $\lambda_{\mathrm{ideal}}$, and estimates the observer's criterion, λ_*obs*_, as the distance from λ_*ideal*_ using the observer's false alarm rate. This formulation is an extension of the standard formula for criterion (Equation 2). Whereas in Equation 2, the ideal criterion maximizes proportion correct at a single signal level, the ideal criterion in Equation 4 accounts for all signal levels.

All analyses were conducted using R software for Statistical Computing (R Core Team, 2013). *d*^′^ and threshold were analyzed with mixed factorial analyses of variance, with group (same vs. different textures) as the between-subject factor, and noise (low vs. high), day (1 vs. 2) and (where relevant) contrast (7 levels) as within-subjects factors. Significant interaction effects were analyzed with simple main effects and *t* tests. Seven subjects (four from the same texture group and three from the different texture group) were excluded from all analyses because performance was at chance for these subjects on one or both of the two days, in one or more of the noise levels. Four of these subjects were at chance performance in both noise levels on both days. Of the 77 subjects included in the analyses, 40 were in the same texture group and 37 in the different texture group. An additional two subjects were removed from the threshold analyses because a reliable threshold could not be calculated in at least one condition on at least one day.

Owing to the presence of outliers, the criterion data were analyzed with nonparametric tests of significance adapted for factorial designs. Factorial permutation tests were conducted using the ezPerm function from the ez package in R (Lawrence, 2016). For all analyses, experiment (Nottingham, McMaster_1, McMaster_2) was included as a factor to determine whether the results were consistent across replications.

### Sensitivity (d’)

**Figure 2.**
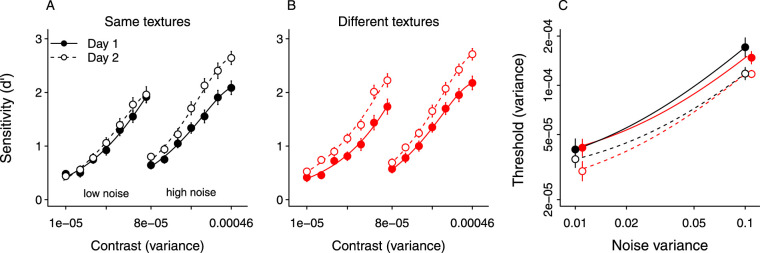
Performance on the texture detection task in two noise levels for both groups on days 1 and 2. Solid symbols: Day 1. Open symbols: Day 2. (A, B): Sensitivity (*d*^′^) plotted as a function of stimulus contrast for the same texture (black; *n* = 40) and different texture (red; *n* = 37) groups. Symbols show mean in each condition, error bars show standard error of the mean. (C): Threshold vs. external noise for both groups (*n* = 39, 36), on days 1 and 2.

Figure 2A and 2B show sensitivity across contrast at each noise level for both groups on days 1 and 2. As expected, *d*^′^ increased with contrast. *d*’ increased from day 1 to day 2 for both groups in all conditions, indicating that both groups improved with practice. In addition, the slope of the psychometric function was greater on day 2 than day 1, although this effect appeared to differ between the low and high noise conditions in the same texture group. Finally, the effect of practice was larger in the different texture group compared with the same texture group in the low noise condition. We first analyzed the data from day 1 only, to confirm that the groups were equivalent at baseline. A 3 (experiment) x 2 (group) × 2 (noise) × 7 (contrast) analysis of variance (ANOVA) revealed that the factor experiment was not significant and did not interact with any of the variables. There was a significant main effect of contrast, *F*(6, 438) = 270.49, *p* < 0.0001, $\eta_{p}^{2}$ = 0.79, and noise, *F*(1, 73) = 37.48, *p* < 0.0001, $\eta_{p}^{2}$ = 0.34, but the main effect of group was not significant, *F*(1, 73) = 0.31, *p* = 0.57, confirming that the two groups did not differ on day 1. The noise × contrast interaction was significant, *F*(6, 438) = 3.71, *p* = 0.001, $\eta_{p}^{2}$ = 0.05, suggesting that *d*^′^ increased with contrast more in high noise than in low noise (i.e., the slope of the psychometric function was steeper in high noise; see Figure 2). The remaining interactions were not statistically significant, *F* ≤ 3, *p* ≥ 0.08 in each case.

Next, we analyzed the data from both days, including day as a factor. Here, the ANOVA revealed a significant main effect of experiment, *F*(2, 73) = 3.54, *p* = 0.033, $\eta_{p}^{2}$ = 0.09. Mean *d*^′^, averaged across noise, contrasts, and day, was 1.38, 1.41, and 1.13 in experiments 1-3, respectively, and pairwise comparisons using Tukey HSD indicated that the difference between experiments 2 and 3 was significant. Experiment did not interact with any of the variables. As expected, the ANOVA found a significant main effect of contrast, *F*(6, 438) = 518.98, *p* < 0.0001, $\eta_{p}^{2}$ = 0.88; noise, *F*(1, 73) = 96.24, *p* < 0.0001, $\eta_{p}^{2}$ = 0.57; and a significant noise × contrast interaction, *F*(6, 438) = 9.61, *p* < 0.0001, $\eta_{p}^{2}$ = 0.12. The main effect of day was significant, *F*(1, 73) = 82.53, *p* < 0.0001, $\eta_{p}^{2}$ = 0.53, because sensitivity was, on average, higher on day 2 than day 1. These main effects were qualified by a significant day × contrast interaction, *F*(6, 438) = 10.59, *p* < 0.0001, $\eta_{p}^{2}$ = 0.13, which reflected the fact that the change in sensitivity across days was greater at high stimulus contrasts than low stimulus contrasts. In addition, there was a significant day × noise interaction, *F*(1, 73) = 4.69, *p* = 0.033, $\eta_{p}^{2}$ = 0.06, which suggests that the change in sensitivity across days was greater in high noise than in low noise. Finally, there was a significant interaction between group, day, and noise, *F*(1, 73) = 5.44, *p* = 0.02, $\eta_{p}^{2}$ = 0.07, which suggests that the difference in improvement between groups depended on noise level. The other interactions between variables were not significant, *F* ≤ 4, *p* ≥ 0.09 in each case.

To analyze the three-way interaction between group, day, and noise, we first averaged *d*^′^ for each subject across stimulus contrasts, and then conducted separate 2 (group) × 2 (day) ANOVAs for each noise level. The group × day interaction was significant in the low noise condition, *F*(1, 75) = 8.32, *p* = 0.005, $\eta_{p}^{2}$ = 0.10, but not the high noise condition, *F*(1, 75) = 0.23, *p* = 0.63. This result confirms the pattern shown in Figure 2, which suggests that in low noise, but not in high noise, the different texture group improved more than the same texture group. We also conducted separate 2 (day) × 2 (noise) ANOVAs for each group: The day × noise interaction was significant in the same group, *F*(1, 39) = 12.32, *p* = 0.001, $\eta_{p}^{2}$ = 0.24, but not the different group, *F*(1, 36) = 0.02, *p* = 0.88. Hence, the same texture group improved more in high noise than in low noise, whereas the different texture group improved by a similar amounts in both noise levels. Overall, these results suggest that both groups improved with practice, and that the different texture group improved more in low noise than the same texture group.

### Contrast thresholds

Figure 2C shows contrast threshold plotted against noise for each group on days 1 and 2: Thresholds were higher in high noise than in low noise, and decreased from day 1 to day 2 for both groups. Log-transformed thresholds from day 1 were analyzed with a 3 (experiment) × 2 (group) × 2 (noise) ANOVA. On day 1, the main effect of noise was significant, *F*(1, 69) = 215.16, *p* < 0.0001, $\eta_{p}^{2}$ = 0.76, because thresholds were generally higher in the high noise condition. There was no significant main effect of experiment, *F*(1, 69) = 1.47, *p* = 0.23, or group, *F*(1, 69) = 0.10, *p* = 0.74, and none of the interactions were significant, *F* ≤ 1, *p* ≥ 0.50 in each case.

Thresholds from both days were analyzed with a 3 (experiment) × 2 (day) × 2 (group) × 2 (Noise) ANOVA. There was a significant main effect of noise, *F*(1, 69) = 429.80, *p* < 0.0001, $\eta_{p}^{2}$ = 0.86, and a significant main effect of day, *F*(1, 71) = 24.31, *p* < 0.0001, $\eta_{p}^{2}$ = 0.26, indicating that thresholds were lower on day 2 than day 1. The main effect of group was not significant, *F*(1, 69) = 0.25, *p* = 0.61, nor was the group × noise × day interaction, *F*(1, 69) = 2.50, *p* = 0.11, $\eta_{p}^{2}$ = 0.004. No other interactions were significant, *F* ≤ 3, *p* ≥ 0.1 in each case. Overall, these results suggest that thresholds of both groups improved by the similar amounts in both noise levels.

### Criterion measures

**Figure 3.**
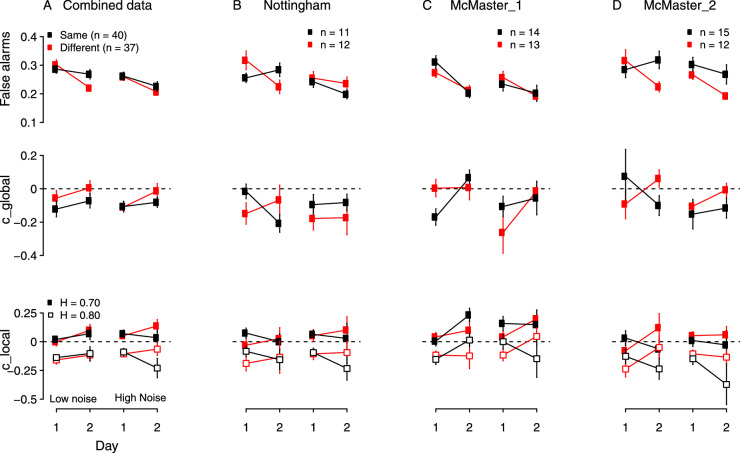
False alarms (mean) and two measures of the criterion (medians) plotted against day, for both noise levels and both groups. (A) Data combined across the three experiments (i.e., replications). (B-D) Experiments 1-3 (Nottingham, McMaster_1, McMaster_2). Top row: False alarms. Row 2: c_global calculated using Equation 4. This measure accounts for all signal levels used in the task. Row 3: c_local at hit rates of 70% and 80%, see Equation 2 and text. Error bars show the standard error of the mean for false alarms, and standard error of the median for the remaining measures, calculated as the standard deviation of the bootstrapped distribution of medians in each condition.

Figure 3 shows mean false alarms and the medians of both measures of the response criterion (Equations 2-6) plotted against day, for both groups and both noise levels. For these measures, the results differed across experiments (replications), therefore the figure shows the results from each experiment separately (columns B-D), as well as all experiments combined (column A).

#### False alarms

False alarms (Figure 3, top row) were analyzed with a 2 (group) × 2 (day) × 2 (noise) × 2 (experiment) ANOVA, which found that false alarms decreased significantly across days, *F*(1, 71) = 48.26, *p* < 0.0001, $\eta_{p}^{2}$ = 0.40, and were higher in low noise than in high noise, *F*(1, 71) = 13.58, *p* = 0.00044, $\eta_{p}^{2}$ = 0.16. The following interactions were significant: group × day, *F*(1, 71) = 9.35, *p* = 0.003, $\eta_{p}^{2}$ = 0.12, group × noise × day, *F*(1, 71) = 4.43, *p* = 0.04, $\eta_{p}^{2}$ = 0.06; group × experiment × day, *F*(2, 71) = 3.98, *p* = 0.02, $\eta_{p}^{2}$ = 0.10; and group × experiment × noise × day, *F*(2, 71) = 8.065, *p* = 0.00069, $\eta_{p}^{2}$ = 0.19. The four-way interaction was decomposed with two separate ANOVAs that examined the effects of experiment, group, and day at each noise level. In low noise, the three-way interaction between group, experiment, and day was significant, *F*(2, 71) = 10.42, *p* = 0.001, $\eta_{p}^{2}$ = 0.23. In high noise, the same three-way interaction was not significant, *F*(2, 71) = 1.22, *p* = 0.29, and only the main effect of day was significant, *F*(1, 71) = 24.34, *p* < 0.001. $\eta_{p}^{2}$ = 0.26.

Figure 3 shows that in low noise, false alarms did not decrease consistently from day 1 to day 2 for both groups across experiments. In experiment 1 (Nottingham) and experiment 3 (McMaster), false alarms decreased for the different texture group but not for the same texture group. In experiment 2 (McMaster), false alarms decreased for both groups. This difference between groups across experiments was confirmed with two additional ANOVAs in the low noise level, showing a significant interaction between experiment and day for the same texture group, *F*(2, 37) = 14.91, *p* < 0.00001, $\eta_{p}^{2}$ = 0.45, but not the different texture group, *F*(2, 34) = 0.637, *p* = 0.53. Hence, the four-way interaction in the combined results arose from the same texture groups in experiments 1 and 3, who disrupted the pattern of a decrease in false alarms with practice.

**Figure 4.**
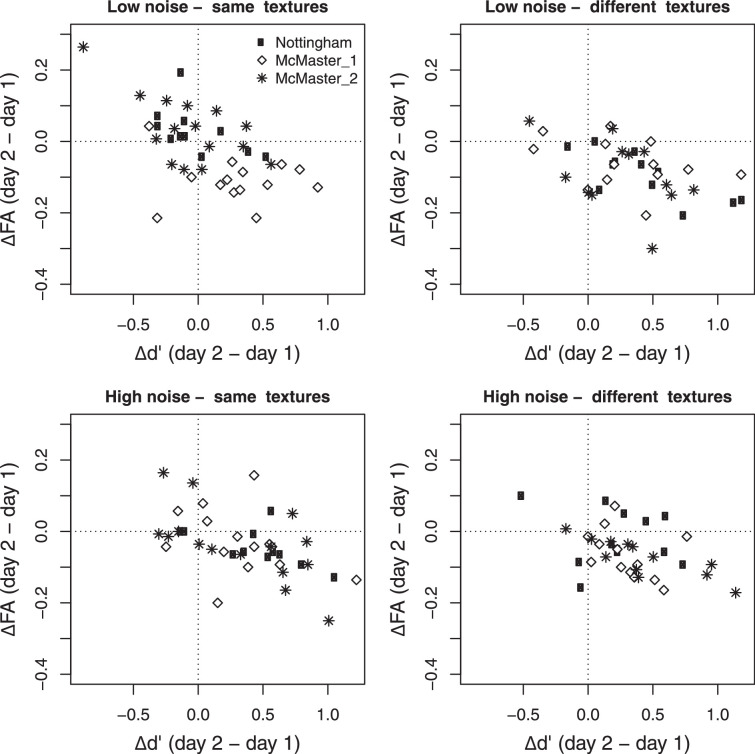
Change in false alarms plotted against change in d’ for each subject. (Top) Low noise. (Bottom) High noise. (Left) Same texture group. (Right) Different texture group. Symbol indicates experiment. Improvement from day 1 to day 2 is shown by points to the right of the vertical dashed line. A decrease in false alarms is shown by points below the horizontal dashed line.

Figure 4 shows the relationship between the change in performance and the change in false alarms for each subject. As expected, changes in false alarms were strongly negatively correlated with the changes in *d*^′^ (Low noise, same texture: *r* = −0.59, *t*(38) = 4.53, *p* < 0.0001; Low noise, different texture: *r* = −0.49, *t*(35) = 3.33, *p* = 0.002; High noise, same texture: *r* = −0.53, *t*(38) = 3.85, *p* = 0.0004; High noise, different texture: *r* = −0.44, *t*(35) = 2.92, *p* = 0.005). Several subjects from the same texture group in low noise did not improve across days, and showed an increase in false alarms (top left quadrant). Consistent with the analyses reported above, these subjects were primarily from experiment 1 (Nottingham; black symbols), and experiment 3 (McMaster, asterisks).

#### c_global

The criterion measures were analyzed using a factorial permutation test that permits evaluation of higher-order interactions (Lawrence, 2016). Figure 3 shows the median c_global calculated using Equation 4. The combined data show that the criterion shifted rightward, from being slightly liberal on day 1 toward an unbiased, or optimal, criterion on day 2 for both groups. A factorial permutation test revealed a significant interaction between experiment, group, noise and day for c_global (*p* = 0.014). The four-way interaction was decomposed with additional permutation tests conducted separately in low and high noise. In low noise, the three-way interaction between experiment, group and day was significant (*p* = 0.008). In high noise, the same three-way interaction was not significant (*p* = 0.87), and none of the other effects were significant.

Figure 3 suggests that the three-way interaction in low noise arose because the criterion did not become less liberal in the same texture group in experiments 1 and 3. This observation was confirmed with separate analyses examining the effects of experiment and day for each group in low noise. For the same texture group, there was a significant two-way interaction between experiment and day (*p* = 0.004). For the different texture group, the two-way interaction was not significant (*p* = 0.358), and there was only a main effect of day (*p* = 0.001). The experiment × day interaction for the same texture group in low noise was further examined with three permutation tests evaluating the effect of day in each experiment. The effect of day was significant in experiment 2 (*p* = 0.001) but not in the other two experiments.

To summarize the pattern so far, in general, groups who showed a rightward criterion shift (i.e., the response criterion became less liberal) were the same groups whose false alarms decreased across days. Hence, the results for c_global resemble those for false alarms.

#### c_local

Figure 3 shows c_local calculated from Equation 2 for hit rates of 70% and 80%. As expected, c_local was more liberal at a hit rate of 80% than 70%. The effect of practice on c_local was in the opposite direction to c_global for the same texture group in high noise (i.e., c_global became less liberal and approached an unbiased, optimal value, but c_local became more liberal). Aside from this difference, the effect of practice on c_local resembled the effects found with c_global. A factorial permutation test revealed that the four-way interaction between group, experiment, noise and day was not significant for $c\_{\mathrm{local}}_{70}$ (*p* = 0.057) or $c\_{\mathrm{local}}_{80}$ (*p* = 0.084). In addition, the group × day interaction was not significant for $c\_{\mathrm{local}}_{70}$ (*p* = 0.054), and $c\_{\mathrm{local}}_{80}$ (*p* = 0.051).

Again, the difference across experiments arose from experiments 1 and 3, where in low noise the response criterion shifted in opposite directions across days in the same and different groups, a result similar to the one obtained with the global criterion and false alarms. In experiments 1 and 3, the same group's criterion decreased across days, whereas it increased (or stayed the same) for the different texture group in all experiments. The main difference between the global and local criterion is that the global criterion appears to become more optimal across days, whereas the local criterion may be interpreted as becoming more or less optimal depending on the hit rate at which it is measured (e.g., compare $c\_{\mathrm{local}}_{70}$ and $c\_{\mathrm{local}}_{80}$ for the same texture group in high noise in the combined data).

In summary, the results combined across the three experiments show that the effect of practice on response bias depended on the measure of response criterion. False alarms decreased with practice, and measures that were based on the hit rates at all stimulus contrasts (c_global) became less liberal and closer to an unbiased, optimal value. However, when a single contrast was used to define the response criterion ($c\_{\mathrm{local}}_{70}$, $c\_{\mathrm{local}}_{80}$) practice caused the criterion to become less liberal in three of four conditions, and more biased according to $c\_{\mathrm{local}}_{70}$. Finally, the effects of practice on response criterion were more consistent across experiments in the high noise condition than the low noise condition.

#### Comparison of global and local measures

**Figure 5.**
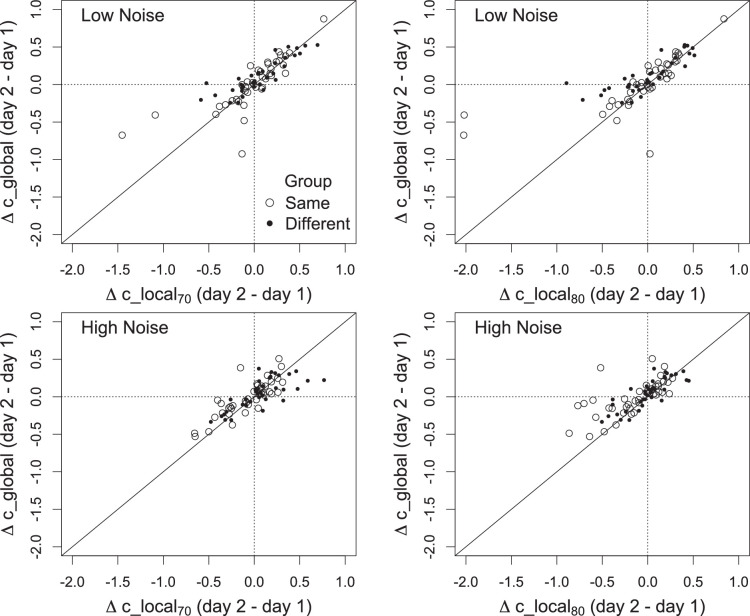
The relation between changes (i.e., day 2 - day 1) in global and local measures of criterion. Changes in global and local measures were positively associated in the the same and different groups and in the low and high noise conditions.

**Figure 6.**
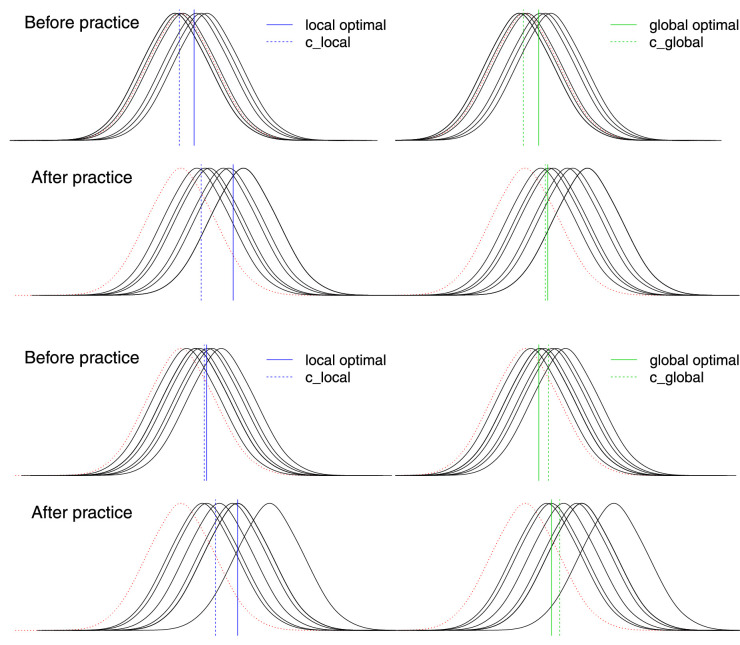
Illustration of practice effects on c_global and c_local for two observers (s1: top two rows, s2: bottom 2 rows). The means of signal (black solid) and noise (red dotted) distributions and the locations of local and global criteria are based on performance measured on days 1 and 2. The optimal local criterion (solid blue) is calculated at a single hit rate (here 80% on day 1). The optimal global criterion (solid green) maximizes proportion correct across the ensemble signal contrasts. The observed values of c_global and c_local are indicated by dashed lines, and response bias is the difference between observed and optimal criteria. For s1, c_local and c_global shift in opposite directions from day 1 to day 2: across days, the local criterion shifts leftward relative to the optimal value (i.e., becomes more liberal) whereas the global criterion shifts rightward (i.e., becomes more conservative). For s2, c_local shifts leftward whereas the c_global is unchanged. See text for more details.

Changes in global and local measures of response criteria that occurred between days 1 and 2 were positively correlated in both the same and different textures groups and in the low and high noise conditions (Figure 5). Generally speaking, a conservative or liberal shift in c_global was accompanied by a shift in the same direction of c_local. Nevertheless, examination of Figure 5 reveals that global and local criteria occasionally changed in opposite directions. This result also is evident in Figure 3: Although false alarms decreased across days in the high noise condition, the median values of c_global increased whereas the median values of c_local decreased (particularly in the same group). This change in opposite direction is possible because the value of c_global depends on the hit rate at every signal contrast whereas c_local depends on the hit rate at only one contrast. Figure 6 illustrates how this occurred in two observers. The figure shows the noise and signal distributions for each observer based on their performance on days 1 and 2. The optimal local criterion is calculated at a single hit rate (here, 80% on day 1, and the corresponding hit rate at the same signal contrast on day 2). The optimal global criterion is calculated across the ensemble of hit rates on each day. The observed values of c_global and c_local are based on each of these optimal criteria. The same false alarm rate is used for c_local and c_global, so the location of both measures relative to the noise distribution is the same (note that the blue and green dashed lines are in same location on each day). The difference between the local and global measures is associated with their positions relative to the optimal values. Practice produces different effects on c_global and c_local when the optimal criteria differ. For s1, c_local and c_global shift in opposite directions from day 1 to day 2 because the local optimal criterion is to the right of the global optimal on day 2. For s2, c_local shifts leftward and c_global is unchanged, again because the local optimal is to the right of the global optimal on day 2.

## Discussion

We found that sensitivity in a yes-no detection task increased across days regardless of whether observers performed the task with the same or different textures on day 2 (Figure 2). Thus, learning in a detection task generalized to novel textures. This result differs from previous studies showing stimulus specific improvements in detection of simple patterns (Dorais & Sagi, 1997; Sowden et al., 2002), and stimulus specific learning in texture identification tasks (Hussain et al., 2009). The results are compatible with previous work showing that improvements in contrast sensitivity generalize across grating orientation (Mayer, 1983; Sowden et al., 2002), although those experiments measured luminance contrast thresholds in the absence of noise. Overall, our results suggest that improvements in detection of textures are not driven by observers becoming more efficient at encoding particular stimulus features. Instead, our results suggest that observers may become more sensitive to the general spatial characteristics of the stimuli. For example, they may simply be better at detecting contrast energy within the 2 to 4 cy/image bandwidth that defined our textures. If this is the case, then it is possible that learning would not generalize to textures constructed from a different spectral band (e.g., 8-16 cy/image).

Improvements in sensitivity from day 1 to day 2 were associated with decreases in false alarms. Observers who did not improve showed either no change in false alarms, or an increase in false alarms across days (Figure 4). This result contrasts with Wenger and Rasche (2006), who reported that subjects who improved on a yes-no contrast detection task showed *increases* in false alarms, and a leftward (liberal) criterion shift, as measured by a local criterion at a hit rate of 79%. Jones et al. (2015) used an auditory amplitude-modulation yes-no detection task and found instead that the effects of practice on response bias depended on whether sensitivity at all signal levels was accounted for in the criterion measure. For their subjects, bias as measured by Wenger's (2006) local criterion increased with practice (subjects became more liberal), but bias decreased when measured by the global criterion. Consistent with Jones et al. (2015), we found that, combined across all experiments, the global criterion for both groups became more optimal with practice, although this was a small effect and not significant in all conditions. It is not clear why false alarms increased in Wenger and Rasche (2006), unlike what we found here. The discrepancy between studies is unlikely to be due to differences in method or experimental design, because Wenger and Rasche (2006), Jones et al. (2015) and ourselves all tested yes-no detection using multiple signal levels presented with method of constant stimuli, and signal absent trials interleaved within the same block (i.e., one false alarm rate for all signal levels). It is worth noting here that the effects of practice on bias may differ for adaptive methods and in nAFC tasks.

The local criterion for our observers moved in the same direction as the global criterion in most conditions (except for the same texture group in high noise), and its overall position depended on hit rate (i.e., relatively conservative at a hit rate of 70% and relatively liberal at a hit rate of 80%). Whether the local criterion became more or less optimal depended on the hit rate at which it was calculated. For instance, in the combined data, the same texture group's criterion in the high noise condition moved leftward at hit rates of both 70% and 80%. At a hit rate of 80%, this appears to be a shift toward a less biased, optimal criterion, whereas at 70% bias appears to increase. Therefore, a measure of criterion that accounts for sensitivity at all signal levels may be more useful in situations where multiple signal levels are used.

The effects of practice on performance were more variable in low noise than in high noise, reflected by the variation of the same texture groups’ performance across experiments 1 through 3. For the same texture group, sensitivity in the low noise condition improved less across days in experiments 1 and 3 than in experiment 2, and several observers showed either no change or an increase in false alarms. The criterion measures varied across experiments in the same way as false alarms. On average across experiments and groups, sensitivity was lower in low noise than in high noise (i.e., the task was more difficult in low noise), which may have increased the variability of all response measures (including criterion) in this condition. Individual differences in learning have been reported for other perceptual tasks (e.g., Saffell & Matthews, 2003; Grzeczkowski et al., 2017), and it is not clear whether these individual differences arise from task difficulty or other factors.

## Conclusions

In contrast with learning of visual discrimination and identification, learning of detection of textures involves a flexible strategy that encompasses the general spatial characteristics of the stimuli. The effects of practice on response bias in yes-no detection are better estimated by a measure that accounts for sensitivity at all signal levels used, than by a measure that is calculated at a single performance level.
